# Supplementary material for: Arabidopsis RCD1 coordinates chloroplast and mitochondrial functions through interaction with ANAC transcription factors
Source: eLife. 2019 Feb 15;8:e43284. doi: 10.7554/eLife.43284 (PMC6414205; doi:10.7554/eLife.43284)
Supplement: Figure 7—source data 3. [file elife-43284-fig7-data3.docx]

**Figure 7 – supplemental dataset 3.** NMR constraints and structural statistics for the ensemble of the 15 lowest-energy structures of RCD1 RST.

| **Completeness of resonance assignments**  **(structured region, 512-568/all residues, 462-589)^a^** | | | |
| --- | --- | --- | --- |
|  | Backbone (%) | | 97.1/95.6 |
|  | Side chain, aliphatic (%) | | 94.9/91.3 |
|  | aromatic (%) | | 85.7/65.6 |
| **Experimental restraints** | | | |
|  | Distance restraints | | |
|  | | Total | 1026/1288 |
|  | | Short (i-j≤1) | 580/825 |
|  | | Medium range (1<i-j<5) | 307/314 |
|  | | Long range (i-j≥5) | 140/149 |
|  | Dihedral angle restraints | | 110/110 |
|  | No. of restraints per residue | | 18.0/10.1 |
|  | No. of long-range restraints per residue | | 2.5/1.2 |
| **Residual restraints violations** | | | |
|  | Average no. of distance violations per structure | | |
|  |  | 0.1-0.2 Å | 5.1 |
|  |  | 0.2-0.5 Å | 0.1 (max 0.28 Å) |
|  |  | >0.5 Å | 0 |
|  | Average no. of dihedral angle violations per structure | | |
|  |  | 0-5° | 0 |
|  |  | > 5° | 0 |
| **Model quality^b^** | | |  |
|  | RMSD backbone atoms (Å) | | 0.7 |
|  | RMSD heavy atoms (Å) | | 1.1 |
|  | RMSD bond lengths (Å) | | 0.014 |
|  | RMSD bond angles (°) | | 2.1 |
| **Molprobity Ramachandran statistics^b^** | | |  |
|  | Most favoured regions (%) | | 98.4 |
|  | Allowed regions (%) | | 1.6 |
|  | Disallowed regions (%) | | 0.0 |
| **Global quality scores (raw/Z score)** | | | |
|  | Verify3D | | 0.28/-2.89 |
|  | ProsaII | | 0.39/-1.08 |
|  | PROCHECK(φ-ψ)^b^ | | 0.17/0.98 |
|  | PROCHECK (all)^b^ | | -0.14/-0.83 |
|  | Molprobity clash score | | 1.43/1.28 |
| **Model contents** | | | |
|  | Ordered residues | | 57 |
|  | Total no. of residues | | 128 |
|  | BMRB accession number | | 27034 |
|  | PDB ID code | | 5N9Q |

^a^Backbone includes C’, C^α^, C^β^, C, N and H, except the N-terminal amide. For side chains, excluded are the highly exchangeable groups (Lys, amino, Arg, guanido, Ser/Thr/Tyr hydroxyl, His^δ1/δ2^), as well as all non-protonated carbons and nitrogens. ^b^Ordered residues: 512-568.
